# Item-Level Analysis of a Newly Developed Interactive Nutrition Specific Physical Exam Competency Tool (INSPECT) Using the Rasch Measurement Model

**DOI:** 10.3390/healthcare10020259

**Published:** 2022-01-28

**Authors:** Sunitha Zechariah, Jennifer L. Waller, Judith Stallings, Ashley J. Gess, Leigh Lehman

**Affiliations:** 1 College of Allied Health Sciences, Augusta University, Augusta, GA 30912, USA; jstallin@augusta.edu; 2 Medical College of Georgia, Augusta University, Augusta, GA 30912, USA; jwaller@augusta.edu; 3 College of Education, Augusta University, Augusta, GA 30912, USA; agess@augusta.edu; 4 School of Occupational Therapy, Brenau University, Gainesville, GA 30501, USA; llehman@brenau.edu

**Keywords:** Rasch model, item-level analysis, nutrition-focused physical exam, registered dietitian nutritionists, competency

## Abstract

The Interactive Nutrition Specific Physical Exam Competency Tool (INSPECT) is a tool designed specifically to observe and measure registered dietitian nutritionists’ (RDNs) nutrition-focused physical exam (NFPE) competence in authentic acute care settings. The initial INSPECT items were generated and tested for content and face validity using expert RDNs’ input. The INSPECT was further examined for inter-rater, intra-rater, and internal consistency using clinical supervisor observations of RDNs performing NFPE on patients in real-life acute care settings. These previous studies showed the INSPECT to have excellent content validity, acceptable face validity, good inter-rater reliability, moderate to strong intra-rater reliability, and excellent internal consistency. In the current study, the Rasch measurement model was applied to examine the item-level properties of the INSPECT. Results confirm that the INSPECT measured a single construct. All items fit the established criteria for clinical observations of >0.5 and <1.7, had positive point measure correlations, met the Wright Unidimensionality Index criteria of ≥0.9, exhibited one latent construct with >40% variance explained by the Rasch dimension as well as a sub-dimension based on item difficulty from the principal component analysis of the first contrast Rasch residuals. Rasch rating scale analysis revealed that the rating scale and majority of the items (39/41) fit the Rasch model. Rasch item hierarchy analysis matched the a priori hypothesized hierarchy for the top-most and bottom-most items. Ceiling effects were seen for three items (hand hygiene, personal protective equipment, and patient position) and one item (handgrip using hand dynamometer) reached the floor effect. Rasch reliability assessment demonstrated high person reliability (0.86), high item reliability (0.96), and person separation of 3.56 ability levels. The principal component analysis of residuals revealed two factors based on item difficulty, one for micronutrient exam and another for macronutrient exam, initial steps, and bedside manner. The resulting two factors may likely be due to a sub-dimension of the latent NFPE trait. Overall, the INSPECT items were found to have good item-level psychometrics. Continued testing of the INSPECT with RDNs at different ability levels will help to determine cut-off scores ranging from novice to expert. Establishing cut-off scores for the INSPECT will further enhance the utility of the tool.

## 1. Introduction

The competence of healthcare professionals directly impacts the delivery of safe, effective, and patient-centered care [1,2,3,4,5]. Registered dietitian nutritionists (RDNs) are an invaluable part of the interdisciplinary healthcare team as they are responsible for navigating patients’ complex nutritional needs. RDNs are trained experts in diagnosing patients with malnutrition and other nutrition-related complications. Historically, RDNs have been educated in the use of anthropometrics, biochemical parameters, clinical evaluation, and diet history to assess patients’ nutritional status [6,7,8]. These parameters, although useful, provide limited information to precisely diagnose malnutrition, particularly undernutrition. Therefore, the Academy of Nutrition and Dietetics (the Academy) added the nutrition-focused physical exam (NFPE) to the Standards of Practice (SOP) and Standards of Professional Performance (SOPP), allowing RDNs to utilize NFPE to improve their malnutrition diagnostic skills [9,10,11]. 

During NFPE, the RDNs perform a head-to-toe physical exam that consists of inspection and palpation of various areas of the body to identify muscle wasting, subcutaneous fat losses, fluid accumulation, and the presence of reduced grip strength. The RDNs also examine other areas such as the hair, eyes, mouth, skin, and nails to detect signs of micronutrient deficiencies. The RDNs then assemble all of the patients’ information including the details gathered from patient interviews and medical records as part of a comprehensive nutrition assessment to determine the presence and severity of malnutrition and/or any signs of micronutrient deficiencies [12,13]. Despite the benefits of applying NFPE in nutrition assessments, NFPE was not frequently utilized by most RDNs [7,14,15]. The Academy has highly encouraged RDNs to embrace and employ NFPE in daily clinical practice and has provided hands-on training sessions, video demonstrations, and simulation models to prepare RDNs in performing NFPE [16]. The training from the Academy and other nutrition organizations has led to a gradual increase in the utilization of NFPE by RDNs in routine clinical practice [16]. 

Despite the steady increase in the use of NPFE among RDNs, there is abundant variation in skill and comfort levels while applying NFPE in practice [7,14]. Skill-building and progression toward mastering NFPE competence is an ongoing process requiring regular evaluation in an actual clinical practice setting [12]. Efficient and precise competency tools that allow direct observation and evaluation of RDNs performing NFPE on patients are crucial for monitoring progress in NFPE skill development. Well-designed and validated NFPE competency tools that can be applied in clinical practice settings are severely limited. Recognizing the need to develop and validate an NFPE competency tool, the authors designed the Interactive Nutrition Specific Physical Exam Competency Tool (INSPECT). 

The initial development of the INSPECT began with expert focus group discussions. Seven content and practice RDN experts from the field convened via technology-based focus groups to explore various components required to perform NFPE. The experts identified 70 NFPE items as key components. Using the tool items generated, a preliminary version of the INSPECT was developed with Microsoft Excel^TM^ (2006). The INSPECT tool items were categorized into subsets based on a head-to-toe sequence. The tool was designed to compute subset scores, overall NFPE score, overall percentage, overall total points possible, and overall total items missed. A detailed account of the first phase of the study is given in a previous publication [17].

In the second phase of the study, the preliminary version of the INSPECT was examined by a larger group of 17 RDN practice experts for content and face validation using the Delphi methodology. The experts arrived at a consensus over two Delphi rounds. Face validity of the INSPECT was deemed acceptable with a Cronbach’s α of 0.71, content validity was excellent with an internal consistency of α = 0.97 in the first round and α = 0.96 in the second round, and excellent inter-rater agreement with intraclass correlation coefficient (ICC) of 0.95 for each of the Delphi rounds. Utilizing the expert consensus and their open feedback, a new version of the INSPECT was designed with 41 items. The details of the second phase of the study are outlined in a prior publication [18]. 

The third phase of the study aimed to assess the psychometric properties of the INSPECT using classical test theory methodologies. Clinical supervisors from multi-site acute care hospitals were recruited to use the INSPECT to assess RDNs performing NFPE on patients. Assessment data was collected at time 1 (first assessment) and two weeks later at time 2 (second assessment). Reliability analysis of the INSPECT assessments exhibited good inter-rater reliability (ICC = 0.78 for the first assessment and ICC = 0.68 for the second assessment), moderate to strong intra-rater reliability for 37 of 41 items (Spearman rho = 0.54 to 1.0) and excellent internal consistency (Cronbach’s α = 0.86 for the first assessment and α = 0.92 for the second assessment). Ten out of the 11 INSPECT subsets showed good to excellent internal consistency (α ranging from 0.70 to 0.98). The reliability results support the INSPECT as a reliable tool, which is stable over time and has a good agreement between raters. The full report on the data collection methodology and reliability analysis is presented in a recent publication [19]. 

The current study explores the item-level psychometric properties of the emerging INSPECT using Rasch analysis. The Rasch model offers a rigorous methodology by applying a mathematical approach that directly compares item difficulty and person ability [20,21,22]. This model estimates item difficulty and person ability on the same continuum, making it possible to determine if person ability levels match the difficulty level of the items [23]. More specifically, this study examined assumptions of the Rasch model (i.e., monotonicity, local independence, and unidimensionality) and applied the Rasch model to evaluate item fit, hierarchical order of item difficulty, item-person match, precision with person and item reliability, and separation indices. Principal component analysis (PCA) of the residuals was also analyzed to determine if a secondary dimension exists after accounting for the Rasch derived latent construct. 

## 2. Methods

### 2.1. Study Sample

Fourteen clinical supervisors in acute care hospitals utilized the INSPECT to assess RDNs performing NFPE on patients and provided 57 assessments. The data were examined for missing items and one assessment was eliminated as it had more than 80% missing information. This assessment was not included in the final analysis resulting in a final sample size of 56 assessments (*n* = 56). A detailed description of the study samples and the data collection methods used to obtain the multi-site observational assessments using the INSPECT is given in a recent publication [19]. This study was approved by Augusta University institutional review board (1721423-2). 

### 2.2. Analysis

Rasch analysis was conducted using WINSTEPS Software Version 5.1.1 [24]. For Rasch analysis, the multi-site observational item assessments were coded as ‘complete (4)’, ‘partially complete (3)’, ‘incomplete’ (2), and ‘not applicable’ (treated as missing data). First, the data were examined to determine if the item response theory (IRT) assumptions (monotonicity, local independence, and unidimensionality) were sufficiently met. Following these assumption checks, item fit, hierarchical order of item difficulty, item-person match, precision with person and item reliability, separation indices, and principal components of Rasch residuals were evaluated. 

### 2.3. Rasch Model Assumptions

#### 2.3.1. Monotonicity

A foundational assumption of IRT is monotonicity (i.e., the probability of endorsing a higher/more difficult response increases as person ability increases). This assumption was tested by evaluating the rating scale for each item based on Linacre’s three essential criteria [25]. First, the number of observations for each category of the rating scale was examined to ensure that there were at least 10 observations. This is important to ensure the stability of person ability and item difficulty estimates. Second, the mean ratings for each rating were examined to verify if lower ratings were associated with lower mean person ability and higher ratings with higher mean person ability. Finally, outfit statistics (i.e., mean square residuals (MnSq)) were assessed for each rating category to identify if the response options were being interpreted accurately. These residuals represent the difference between actual ratings and the rating predicted by the Rasch model. Outfit MnSq values >2.0 have been suggested to be of concern [25].

#### 2.3.2. Local Independence

The assumption of local independence is met if responses to each item in the measure are mutually independent of the responses to another item. In other words, the items should only be correlated through the latent construct (e.g., ability to complete NFPE) and should otherwise be independent of each other. If there is a significant correlation among the items after accounting for the latent construct, then the items are locally dependent or there is a secondary dimension of measurement influencing the correlation [26]. Local independence of item pairs was analyzed. Residual correlations >0.30 were considered a concern [27,28].

#### 2.3.3. Unidimensionality

The assumption of unidimensionality is that all items on a given assessment, measure the same underlying latent construct [29]. Unidimensionality was assessed using item fit statistics, point measure correlation, the Wright Unidimensionality Index, and PCA of Rasch Residuals [30,31,32].

### 2.4. Item Fit

The extent to which items fit the theoretical Rasch model was evaluated by examining the infit MnSq and standardized *z* values [27,32]. These residuals represent the observed variance divided by the expected variance (i.e., what is predicted by the Rasch model). Thus, the desired value is one. For clinical observations with rating scales, MnSq of >0.5 and <1.7 are indicative of reasonable mean square fit along with a standardized *z* score of <2.0 in the Rasch model [32,33]. Items with MnSq >0.5 fail to discriminate between people of different abilities or may be redundant. Items with MnSq <1.7 indicate that items may not belong on the same continuum or that the item is being misinterpreted. Items with high MnSq were scrutinized more closely than those with low MnSq as items with high MnSq represent a greater threat to validity. 

#### 2.4.1. Point Measure Correlations

Point measure correlations assess the relationship between real observations and the predicted Rasch measures. Correlations in the positive direction indicate that observations agree with the unidimensional Rasch model [31,34].

#### 2.4.2. Wright Unidimensionality Index

Wright’s Unidimensionality Index represents the person separation index using real standard errors divided by the person separation index using model standard errors. Thus, it reflects how well the observations work together to fit the Rasch model. A value of ≥0.9 is indicative of unidimensionality and ≤0.5 suggests multidimensionality [30].

#### 2.4.3. Principal Components Analysis (PCA)

The PCA of Rasch residuals was utilized to examine the patterns within the data that do not agree with the expected Rasch measures. The PCA aims to first extract the primary (unidimensional) Rasch dimension and then to examine if the remaining residuals contribute to a meaningful secondary dimension or are simply random noise [35,36]. Unidimensionality is considered valid when the Rasch dimension explains at least 40% variance of the observed data and the eigenvalue of the first residual contrast is ≤2.0 [35,36]. The first contrast of the PCA of Rasch residuals can be further examined to identify if the first component in the correlation matrix produces consequential information. Groupings of positive and negative item loadings in the first contrast that conceptually make sense (i.e., if positive loading items reflect something meaningfully different from negative loading items) can be deemed as support for a secondary dimension [32,36]. Although PCA of residuals has been heavily used to indicate unidimensionality, it has been reported as a diagnostic rather than a definitive indicator of secondary dimension [32,36]. In addition, questions have been raised about the accuracy of PCA of residuals in establishing a single dimension as attributes such as sample size can impact the findings [37,38,39]. 

#### 2.4.4. Hierarchical Order of Item Difficulty

Rasch analysis calculates linear item difficulty measures (i.e., logit values) for each item making it possible to evaluate the relative challenges each item presents for the participants [21,40]. Hypothesized hierarchies of difficulty were developed a priori for all items in the INSPECT. It was hypothesized that the easiest item would be ‘hand hygiene (washes/sanitizes hands)’ while the hardest item would be using the ‘handgrip dynamometer’. The hypothesized hierarchy of item difficulty was compared with the Rasch-derived item hierarchy. 

#### 2.4.5. Match between the Items and Person

The items were designed with the intent of measuring a wide range of abilities from novice to expert. The item difficulty and person ability match were investigated to understand how well the items assessed individuals of differing abilities. Ceiling effects, i.e., person abilities exceeding item difficulties, and floor effects, i.e., person abilities lower than item difficulties were identified. Furthermore, for adjacent items, the distance between the lowest possible rating scale choice for the harder item, that is, incomplete, and the highest possible response for the easier item, complete, were examined to determine if any “gaps” were present. These “gaps” prohibit differentiation of individuals whose ability level falls at the place where the gap exists. Gaps were calculated using the formula, *t* = (A − B)/√SE_A_^2^+ SE_B_^2^), where A and B represent the two item calibrations at the bottom category of the harder item and the top category of the easier item and SE_A_ and SE_B_ represent the standard errors of A and B, respectively [29]. Values of *t* greater than 1.96 indicate a significant gap. Ceiling effects, floor effects, and/or gaps suggest that the INSPECT is limited in its ability to precisely measure the abilities of some individuals (i.e., those in the ceiling/floor or who have abilities where gaps exist). 

#### 2.4.6. Precision, Person and Item Reliability, and Separation Indices

Precision was determined using the person and item reliability indices and person-separation reliability ratio. The person reliability index represents the reproducibility of the rater observations whereas the item reliability indicates the consistency of items. Person and item reliability are analogous to Cronbach alpha, where a higher value indicates greater reliability. The reliability indices were interpreted with values ≥0.5 regarded as adequate, ≥0.80 as good, and ≥0.90 as high reliability [22,32].

The separation ratio (SR) was used to investigate the INSPECT’s level of precision. This ratio is defined as the ratio of the standard deviation of the sample in logits adjusted due to error to the standard error of measurement. It is calculated using the formula (4Gp + 1)/3 where Gp is the person separation index [29,32]. A low person separation of <2.0 with a person reliability of <0.8 would indicate that the INSPECT may not be sufficiently sensitive to distinguish between high and low performers. Low item separation of <3.0 and item reliability of <0.9 may indicate that the sample size may be insufficient to confirm the item difficulty hierarchy [32]. A person-separation ratio of >2.0, representing a Rasch reliability of 0.8 was deemed acceptable for this study [32,40,41].

## 3. Results

The INSPECT evaluations of 56 RDNs were used in Rasch analysis. The majority of the RDNs worked as clinical dietitians (*n* = 45, 80.4%) in the inpatient area of acute care hospitals (*n* = 50, 89.2%) with a mean clinical dietetic experience of 5.5 years (±6.6), and a mean NFPE practice experience of 3.3 years (±2). Demographic characteristics of RDN performers are presented in Table 1.

### 3.1. Rasch Model Assumptions

#### 3.1.1. Monotonicity

All three essential criteria were met for use of the Rasch model. All four rating scale categories showed well above 10 observations. For the INSPECT items, a rating of ‘2 = incomplete’ was observed 479 times, a rating of ‘3 = partially complete’ was observed 270 times and a rating of ‘4 = complete’ was observed 1396 times. The mean rating measures revealed that lower ratings were associated with lower person ability and higher ratings with higher person ability. The rating of ‘2’ was associated with a mean person ability estimate of −0.62, a rating of ‘3’ with a mean person ability of 0.07, and a rating of ‘4’ with a mean person ability of 2.07. The outfit MnSq residuals ranged from 0.93 to 1.36, and hence met the criterion of <2.0 [25].

#### 3.1.2. Local Independence

The assumption of local independence was violated as all residual item correlations were found to be >0.32. The premise of local independence in the Rasch analysis is that the items are independent of each other and that a response to one item does not impact the response to another item. However, it has been reported that if a tool has subsets as in the case of the INSPECT, that could potentially affect the local independence as the measure does not describe the behavior of base-level items, but rather describe parameterized subset scores [42]. In addition, some degree of local dependence is expected in empirical data such as the direct clinical observations of the INSPECT. Hence, violation of this assumption should be interpreted conservatively with relevance to the clinical application [43].

#### 3.1.3. Unidimensionality 

Analysis of unidimensionality showed that all of the INSPECT items measured a single domain within the NFPE construct. Unidimensionality was assessed using item fit statistics, point measure correlation, the Wright Unidimensionality Index, and the PCA of Rasch residuals and all of these are outlined below in Section 3.1.4, Section 3.1.5, Section 3.1.6, Section 3.1.7.

#### 3.1.4. Item Fit

The infit MnSq of the INSPECT ranged between 0.5 and 1.7, meeting the established criteria for clinical observations. This supports the interpretation that the items belong to the same continuum [22,32]. The standardized *z* score (ZSTD) of <2.0 was met for 39 of 41 items in the Rasch model [33]. Two items, handgrip subjective measure and skin exam of lower extremities did not meet the ZSTD criterion and were >2.0. One item (handgrip using hand dynamometer) reached floor effect and three items (hand hygiene, personal protective equipment, and patient position) reached ceiling effect. The item fit analysis is shown in Table 2.

#### 3.1.5. Point Measure Correlations

Point measure correlation showed all of the INSPECT items were positive and moved in one direction within the construct (Table 2). Index in the positive range indicates that the measured INSPECT items are parallel to the measured NFPE construct indicating unidimensionality [34,35].

#### 3.1.6. Wright Unidimensionality Index

The Wright Unidimensionality Index for the INSPECT was 0.9, which is ≥0.9 supporting the unidimensionality of the INSPECT items [30].

#### 3.1.7. PCA of Rasch Residuals

PCA of Rasch residuals explained 56% of the observed measures compared to the expected measure of 54.4%. The Rasch model predicts that there will be explained and unexplained variance within all measures. The explained or expected measure corresponded to the primary Rasch dimension within the INSPECT and the unexplained variance paralleled to other variance or random aspects of the data [36,44,45]. The randomness of the data was explored through the first contrast of the PCA of Rasch residuals. The first contrast showed >2.0 eigenvalue. Although the high eigenvalue of the first contrast is of concern, there are several reasons why this does not violate the assumption of unidimensionality. First, the PCA of Rasch residuals is for diagnostic purposes as mentioned before rather than for definitive conclusions of unidimensionality [36,44]. Second, the cut-off points are arbitrary and depend on the sample size and the number of items [46]. Third, as the INSPECT consists of several items grouped as subsets or subcomponents, there is potential for items within these subtests to be more correlated causing the increase in eigenvalue in the first contrast. Fourth, missing data can diminish the utility of PCA Rasch residuals diagnostics [35]. The PCA of Rasch residuals first contrast plot was examined for any meaningful traits and is elaborated later in the results section. 

### 3.2. Rasch Measurement Analysis

#### 3.2.1. Hierarchical Order of Item Difficulty

**Figure 1:**
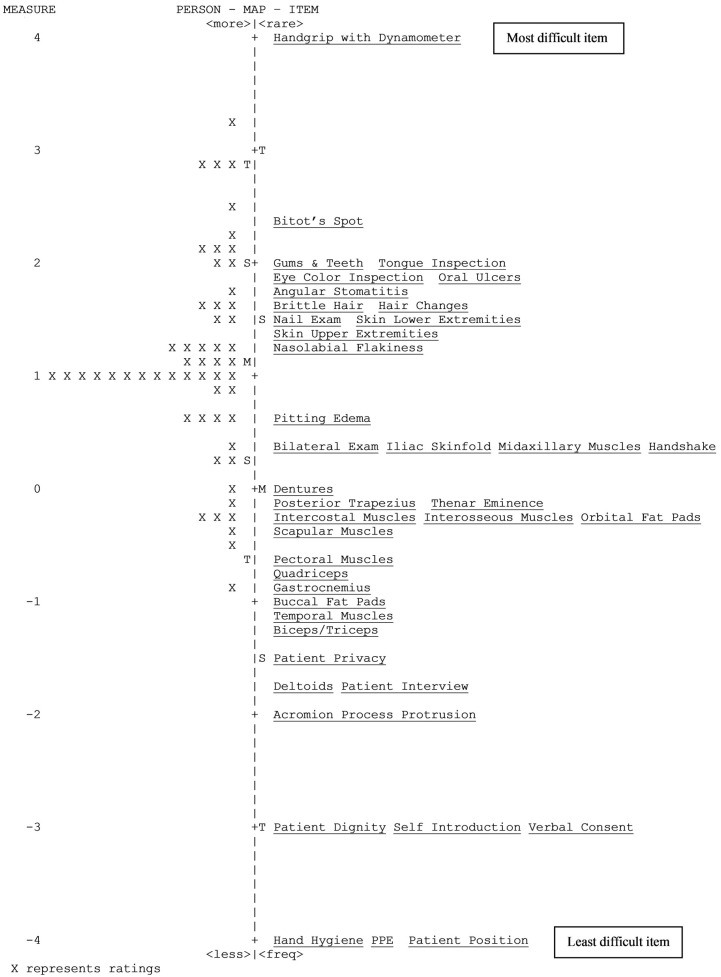
Map of Person Ability and Item Difficulty.

The hierarchical order of item difficulty matched the a priori hierarchy of difficulty for the top-most item and the bottom-most item. The hardest item was the handgrip assessment using the ‘hand dynamometer’ while the easiest item was ‘hand hygiene (washes/sanitizes hands)’. The hierarchy of item difficulty of the INSPECT is displayed in Table 3 and Figure 1 in descending order with the top-most item being the hardest item. Only the top-most item and bottom-most item matched the hypothesized hierarchy while the remaining items did not match. Although the Rasch-derived item hierarchy did not correspond to most of the hypothesized hierarchy, the Rasch hierarchical order was plausible, as RDNs appear to have difficulty performing micronutrient exam items. All of the 12 micronutrient exam items had higher item difficulty estimates compared to the 20 macronutrient exam items, five preparation and initial steps items, and four bedside manner and etiquette items.

#### 3.2.2. Match between the Items and Person

The item-person match allows for direct comparison of item difficulty with person ability. This is illustrated in the Wright map of person ability and item difficulty (Figure 1). The item-person map presents the difficulty measures plotted against person measures at each logit value. The items are located on the right of the map at their average measure (item’s logit value at the middle category of the rating scale). In general, the items were distributed about the mean. The mean person measure was 1.12 logits (standard error = 0.12) above the item calibration mean which was anchored at zero. This indicates that most items were reasonably easy for the performers.

The gap computation showed *t* ranging from 0.42 to 11.8. Thus, there were significant gaps (where *t* was greater than 1.96) between item difficulty estimates. Significant gaps within the INSPECT may indicate certain person ability levels are not being measured precisely or there may be insufficient items to measure different ability levels between the gaps. Items pairs with gaps between them are presented in Table 4. 

#### 3.2.3. Precision, Person and Item Reliability, and Separation Indices

The Rasch person separation value for the INSPECT was 2.42, meeting the criterion of >2.0 [40,41]. The person separation ratio was 3.56, indicating that the INSPECT was able to divide performers into slightly more than three groups based on their ability levels. The Rasch person reliability of the INSPECT was 0.86 and Kuder Richardson-20 analogous to Cronbach’s α for the INSPECT was 0.96.

The Rasch item separation value for the INSPECT items was 4.67, well over the criterion of >3.0 and the item reliability was 0.96, meeting the >0.9 item reliability criterion [24,32]. Results of the precision and reliability indices are presented in Table 5. 

#### 3.2.4. PCA of Rasch Residuals Plot

**Figure 2:**
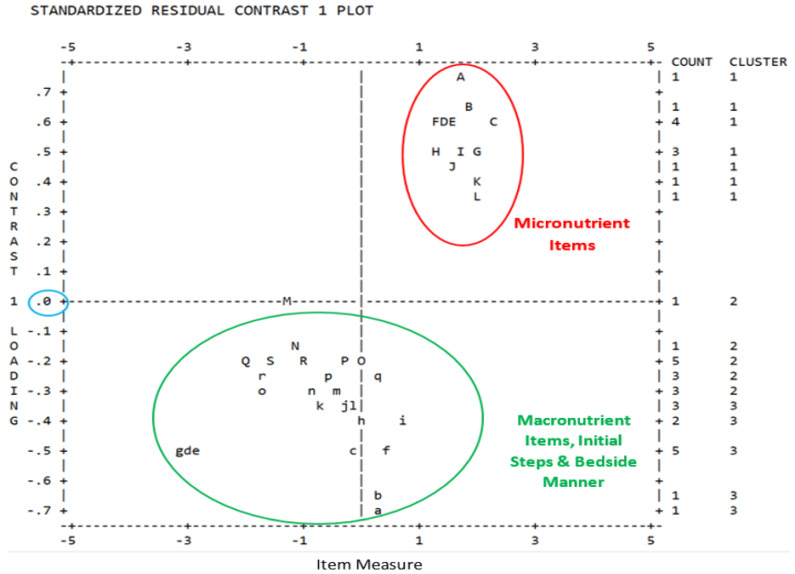
Principal Component Analysis of Rasch Residuals Plot.

Observed PCA residuals explained 56% of the variance while expected residuals explained 54.4%. The eigenvalue of the unexplained variance in the first contrast was 7.05 (8.4%). This translates to 3.4 items out of 41 items. As discussed previously, although a high eigenvalue was seen, there is large merit for one dimension with the INSPECT as there is one rating scale for all items and the items are all of the same dimension, i.e., various parts of the human body where NFPE is performed. In order to understand the variance in the first contrast, a standardized residual first contrast plot was examined to identify if there are clusters of items at the top (positive loading) or bottom (negative loading) of the plot that shared some meaningful traits [36]. Analysis of the PCA plot confirmed the possibility of ‘subdimensions’ caused by INSPECT subsets or subcomponents (i.e., items that fall in the same category are grouped). The plot showed a two-factor solution, which is shown in Figure 2. The first factor had 12 items, all pertinent to the micronutrient exam and the second factor had 25 items with all items applicable to the macronutrient exam, preparation and initial steps, and bedside manner and etiquette. Four items were not able to be measured as they reached either the ceiling or the floor effects. Handgrip with hand dynamometer measure reached floor effect while hand hygiene, personal protective equipment, and the patient position reached ceiling effect. 

## 4. Discussion

Since the competence of healthcare professionals significantly affects patient clinical outcomes and safe patient care, it is vital to have a well-constructed, reliable, and valid tool to evaluate healthcare providers’ ongoing abilities [1,2,3,4,5]. Since NFPE is a recently acquired skill for RDNs, it is critical to develop a tool to appraise RDNs’ competence in performing NFPE during patient assessments. The INSPECT is a competency tool specifically designed to meet this need. The INSPECT was developed and examined over multiple phases that included item generation via focus groups with experts [17], content, and face validation of the preliminary version of the INSPECT utilizing the Delphi methodology [18], and evaluation of the tool as a whole using classical test theory methodologies [19]. The current study examined the item-level psychometric properties of the INSPECT using the Rasch model IRT methodologies.

Initially, the Rasch model assumptions were tested. Investigations for monotonicity showed that the items on the INSPECT met the three essential criteria indicating that as the person ability increased, the ratings on the rating scale also increased, suggesting that the rating scale was being interpreted accurately. Rating scale analysis indicated that the lower rating measures were associated with lower person ability and higher ratings with higher person ability. The mid-rating of ‘3’ did not emerge as a category of its own, however, this rating was introduced in the INSPECT based on the expert input during content validation [18]. As this study only contained RDN performers with a mean clinical experience of 5.8 years, all other ability levels were not examined, which possibly excluded the rating of ‘3’ from emerging as a category. Replicating this study with a sufficient sample size of varying ability levels including dietetic students, dietetic interns, and RDNs with a wide range of NFPE practice experience (experts) might allow this mid-level category to become an additional rating scale on the INSPECT. 

Evaluation of item independence presented a challenge as the items on the INSPECT showed significant correlations between several item pairs, indicating possible local dependence [43]. The violation of independence could possibly be due to the behavior of item subsets rather than base-level items [42]. The INSPECT is designed in subsets, that is, items belonging to a category are grouped into one subset. For example, items belonging to the face exam are grouped into one subset. Similarly, items for the mouth exam are grouped into their own subset. Altogether, the INSPECT has 11 subsets with a varying number of items within each of these subsets. Hence, there is a possibility that the set of items within a subset may share some features that are probably behaving similar to the latent trait but are not being adequately modeled at the item level to reveal local independence [42]. In addition, Baghaei proposes that a degree of local dependence always exists in empirical data [43]. As the INSPECT data are observed assessments from real-life clinical settings, it is likely that some item dependence is possible. Conceptually, as the performance of one item on the INSPECT does not depend on the performance of another item, it can be assumed that this violation is probably due to the INSPECT design rather than the actual behavior of the items [42,43]. 

The assumption of unidimensionality was supported by the item infit statistics meeting the established criteria for clinical observations, the point measure correlations for all items moved in the same positive direction, met the ≥0.9 criterion for the Wright Unidimensionality Index, and the Rasch dimension explained >40% variance of the data indicating the items belonged to the same NFPE construct [31,32,34,35]. The single dimension of the NFPE construct was also confirmed during the content validation phase of the study as 94% of expert participants agreed that the tool intended to measure the competency of RDNs’ NFPE performance [18]. Although the first contrast of PCA of Rasch residuals showed high variance, the reasons discussed in the results section adequately explain why the assumption of unidimensionality is reasonable and acceptable. In addition, the two factors exhibited by the residual analysis are more likely a subdimension of the latent NFPE construct rather than a distinct trait not related to NFPE.

The hierarchical order of item difficulty analyzed using the Rasch model matched the a priori hypothesized hierarchies of difficulty for the top-most and bottom-most items. As predicted, hand hygiene with washing or sanitizing hands was expected to be the first task an RDN performs before starting the physical exam on a patient and hence it was hypothesized as the easiest item. Similarly, many RDNs may not be familiar with operating a hand dynamometer using standardized measurement techniques and hence this item was hypothesized to be the hardest item on the INSPECT. It is not surprising that the objective measure of grip strength using a hand dynamometer was found to be the most difficult item. This finding has been previously reported and was also confirmed during the content validation phase of the study, where experts indicated low usage of hand dynamometers among RDNs and unfamiliarity with standardized measurement techniques in operating the dynamometer [18,47]. In addition, all 12 micronutrient exam items ranked with higher difficulty while the items pertaining to the macronutrient exam ranked with lower difficulty. Again, this is not a surprising finding since most RDNs are familiar with macronutrient exam aspects of NFPE and are hesitant to perform the micronutrient exam parts [13]. This result was evident during the content validation phase as content experts agreed on only 24 of 31 micronutrient exam items [18].

Comparing the item difficulty with person ability on the item-person map indicated that most items were reasonably easy for the current participants. Ceiling effects and floor effects were seen for item measures. One of the items ‘handgrip using hand dynamometer’ reached floor effect, and three items ‘hand hygiene’, ‘patient position’, and ‘personal protective equipment’ reached ceiling effects. The misfit of these items could possibly be a result of variation in responses to these items by the RDN performers. For example, hand dynamometers may not be available in the facilities participating in the study or the RDNs may not have the knowledge to perform the measurement on patients and hence reached floor effect. Likewise, items ‘hand hygiene’, ‘patient position’, and ‘personal protective equipment’ are possibly easy items that are performed by all RDNs and hence reached the ceiling effect. Significant gaps between items were noted, which may mean RDN performers at certain ability levels may not be measured or there may be insufficient items to measure different ability levels. Item gaps identified may suggest that the INSPECT has limitations in differentiating performers’ abilities adequately. Therefore, future studies with different levels of RDN performers, students, and interns should be explored to close the gaps. 

Although the INSPECT did not perfectly match the Rasch model metrics, all of the items within the INSPECT were retained. The decision to retain the items was based on the clinical importance and application of these NFPE items for accurate diagnosis of macronutrient and micronutrient deficiencies. Additionally, eliminating these NFPE items would compromise the content and face validation established previously through practice experts [18]. Furthermore, since RDNs are actively learning the NFPE skill, eliminating items based on PCA residuals to fit the Rasch model may jeopardize the ability to gauge the RDNs’ learning progression and to plan appropriate instructional training [48]. Omitting certain NFPE items may also give the impression to RDN performers that certain areas of the physical exam can be skipped. This may have serious consequences as some areas of deficiency may be missed in patients resulting in an inaccurate diagnosis of macronutrient or micronutrient deficiencies. Moreover, it may become difficult for clinical supervisors to observe and identify the frequently missed NFPE areas, which in turn will hinder the appropriate development of individualized training plans for RDNs. 

The precision analysis to separate performers into various ability levels showed that the INSPECT was sufficiently sensitive to distinguish between high and low performers and was able to divide the performers’ ability into a little more than three ability levels. The Dreyfus model adopted by the Academy distinguishes the ability levels in dietetics practice into five categories: dietetic students as ‘novice’, dietetic interns as ‘advanced beginners’, RDNs at the entry-level as ‘competent’, RDNs with ≥3 years of experience as ‘proficient’, and those well recognized in their area of practice as ‘experts’ [49]. Based on this model, it is likely that the ability of the RDN performers was between competent to the proficient level since students and dietetic interns were not included in this study. 

The Rasch reliability indices resulted in high person reliability and high item reliability. High person reliability indicates that the clinical rater observations of the INSPECT collected from multi-site acute care hospitals were reproducible. This finding confirms our reliability results achieved during the classical test theory analysis of this study [19]. High item reliability suggests that the INSPECT items had high internal consistency, again confirming the results from the classical test theory analysis phase of the study [19]. 

The PCA of Rasch residuals divided the items into a subdimension based on the difficulty level of the exam being performed [32]. The factors clustered into items loading with 12 micronutrient exam items (factor 1 with positive loading) and 25 items loading with macronutrient exam items along with items for the initial steps and bedside manner (factor 2 with negative loading). Factor 1, that is the micronutrient exam items allow RDN NFPE performers to detect signs of essential vitamin and/or mineral deficiencies in patients [13,50]. Intake of micronutrients has been reported to be low among the general population of the United States and globally [50]. Low intake of micronutrients can result in micronutrient inadequacies (intake slightly lower than recommended dietary intake) or micronutrient deficiencies (intake well below the dietary intake recommendations) [51]. While micronutrient deficiencies cause clinically overt signs, micronutrient inadequacies may cause hidden symptoms such as general fatigue, impaired cognition, reduced immunity, etc., which may be difficult to detect clinically [52,53]. Careful evaluation through NFPE, therefore, becomes imperative to determine the overt and covert signs and symptoms of micronutrient inadequacies and deficiencies. The 12 micronutrient exam items on the INSPECT (hair change, brittle/dull hair, eye inspection for color, Bitot’s spot, nasolabial folds, angular stomatitis, gums and teeth, oral ulcers, tongue inspection, nail exam, skin exam of upper extremities and skin exam of lower extremities) would assist the RDNs in evaluating common areas of the body where clinical manifestations of micronutrient deficiencies are evident. 

Factor 2 comprised of macronutrient exam items and items related to initial preparation and bedside manner. Twenty macronutrient exam items with the INSPECT enable RDNs to identify signs of macronutrient deficiencies in the form of muscle loss, subcutaneous fat loss, fluid accumulation, and reduced handgrip strength and relate the findings to normal, moderate, or severe protein-calorie malnutrition [9,54]. As the prevalence of malnutrition is estimated as high as 30% to 50% among patients hospitalized in the United States, RDNs play a significant role in the diagnosis of malnutrition and provision of appropriate treatment to alleviate the condition [6,55,56]. The other five items loaded with factor 2 allow the RDNs to prepare and conduct NFPE appropriately at patients’ bedside. One possible reason that the five items loaded with the 20 macronutrient items could be due to difficulty levels similar to the macronutrient items. Four items did not load due to ceiling or floor effects. 

Overall, the INSPECT exhibited acceptable item-level psychometric properties. Item hierarchy and person ability results provide valuable information regarding the areas where RDN NFPE skills are lacking and allow an opportunity to design training programs needed to improve these NFPE skills. The item-person map indicated that items were generally well-spaced, although there were instances of clustering of items and gaps between items. The INSPECT showed precision, single dimension, and sufficient evidence for construct validity. Given the insight from this study, the authors perceive that the INSPECT can be used as a competency tool to gauge RDNs’ NFPE competence in acute care settings. 

## 5. Strengths and Limitations

A major strength of this study is the use of a mixed-methods approach with rigorous methodologies over multiple phases to design an NFPE competency tool (INSPECT) and to conduct validity studies. Another strength is that this study utilized input from a representative sample of expert RDNs and assessments of RDN NFPE performers from multi-site authentic acute care settings of various geographical regions of the United States. Another major strength is the application of the Rasch model in this phase of the study, which allowed for the exploration of how individual items interacted toward the ability of RDN performers. However, there are limitations to this study. Two major limitations of the study are the misfit of four items and the violation of the local independence assumption. Although, there is reasonable evidence to consider that the local dependence seen was due to the INSPECT design with subsets, additional diagnostic procedures and use of different IRT models may offer additional perspective into the behavior of the items. Another limitation of the study is that the ‘not applicable’ rating was treated as missing data, which could possibly have influenced the results. The INSPECT was assessed only in adult acute care areas, limiting its use in the pediatric population and other settings such as long-term care, rehabilitation, and outpatient clinics.

## 6. Future Research

Future studies that include a larger sample size with different ability levels based on the Dreyfus model may help to better fit the items, may meet the local independence assumption, and may elucidate additional insight into how the INSPECT items relate to different performers’ levels. This would help in creating cut-off scores that distinguish the RDN competence into appropriate ability levels, which in turn would provide the opportunity to expand the use of the INSPECT. Additional studies should be conducted outside of acute care to gauge if the INSPECT sufficiently meets the IRT properties in other types of clinical settings.

## 7. Conclusions

The findings from this study showed that the INSPECT was sufficiently unidimensional for meaningful measurement of NFPE competence. The results also showed that the INSPECT was able to accommodate a wide range of person ability and item difficulty. These findings suggest that the INSPECT can be used for monitoring and documenting RDNs’ competence in performing NFPE in acute care settings. The INSPECT can be a valuable tool to identify competence deficiencies and to design personalized instructional training for continuous improvement in RDNs’ NFPE practice skills. Future studies that test the INSPECT among RDNs with different ability levels to determine cut-off scores ranging from novice to expert will enhance the value of the tool.

## Figures and Tables

**Table 1:** Demographic Characteristics of RDN Performers.

Variable	Mean (SD)	*n* = 56	%
**Primary Job Role**			
Clinical Dietitian		45	80.4
Clinical Dietitian Specialist		8	14.3
Lead Dietitian		3	5.4
**Primary Work Area in Acute Care**			
Inpatient		50	89.2
Outpatient		1	1.8
Critical Care		5	8.9
**Highest Degree Earned**			
Bachelor’s		30	53.6
Master’s		26	46.4
**Years of Practice as Clinical Dietitian**	**5.8 (6.6)**		
**Years of Experience in Performing NFPE**	**3.3 (2)**		

**Table 2:** INSPECT Item Fit.

INSPECT Items	Item Performance Indicators	INFIT		Point Measure Correlation
		MnSq *	ZSTD ^	
Orbital Fat Pads	Inspects and gently palpates orbital fat pads bilaterally	1.52	1.93	0.43
Patient Privacy	Draws curtains, maintains patient’s privacy at all times	1.66	1.25	0.25
Handshake and/or Grip/Squeeze Fingers (subjective measure, not part of Academy/ASPEN diagnostic criteria)	Assesses handgrip strength through handshake and/or asking patient to squeeze examiner’s pointer and middle fingers bilaterally (subjective measure, not part of Academy/ASPEN diagnostic criteria)	1.63	2.63	0.49
Leg Exam for Petechiae and/or Purpura	Inspects skin for small, pinpoint round skin hemorrhages and/or reddish-purple rash on knees, thighs, or legs	1.34	2.04	0.54
Acromion Process Protrusion	Inspects for prominent protrusion of acromion process	0.75	−0.17	0.19
Muscles Around Midaxillary Line	Inspects and palpates muscles around the midaxillary line	1.29	1.46	0.51
Temporal Muscles	Inspects temporalis muscle directly from the front or as feasible for hollowing/scooping, palpates bilaterally using fingers with patient clenching teeth if able	0.97	0.06	0.31
Buccal Fat Pads	Inspects cheeks bilaterally standing directly from the front or as feasible, palpates fat pads under the cheekbones	1.38	1.06	0.34
Intercostal Muscles	Inspects for concave shape and palpates between ribs for depression	1.0	0.06	0.46
Biceps/Triceps	Asks patient to bend the arm at a 90-degree angle, gently grabs biceps/triceps between thumb and pointer finger and rolls to determine the amount of fat	1.33	0.84	0.29
Dentures	Asks if the patient wears dentures and if yes, asks patient if dentures are ill-fitting	1.25	0.96	0.49
Pitting Edema	Inspects and palpates by pressing down on skin over the pretibial area, ankles, and/or foot for 5 s and releases to assess size and depth of the pit	1.25	1.60	0.52
Bitot’s Spots	Using penlight inspects conjunctiva bilaterally for small, foamy, dry, oval, or triangular spots	1.03	0.21	0.49
Posterior Trapezius Muscles	Inspects and palpates trapezius on the back bilaterally	0.94	−0.21	0.46
Hair Changes	Asks about any recent hair changes, too much hair falling out on the pillow or in the shower?	0.96	−0.18	0.53
Gastrocnemius Muscles	With the patient’s leg propped up, gently grasps calf muscle, and assesses for bulk or thinning, may ask the patient to flex toes to engage muscle	1.16	0.58	0.36
Quadriceps Muscles	With patient’s leg propped up and slightly bent at the knee, inspects for concave depression, palpates quads bilaterally	1.13	0.51	0.37
Iliac Crest Prominence & Iliac Crest Skinfolds	Inspects tip of the hip bone for prominent iliac crest, pinches skin between the iliac crest and last rib, and rolls to determine the amount of fat to assess fat loss	0.94	0.26	0.49
Pectoralis Major	Asks patient to sit up if able with arms at sides, inspects directly from the front or as feasible, palpates muscles right below clavicles	1.05	0.28	0.40
Bilateral Inspection & Palpation	Examines patient bilaterally where applicable	0.87	−0.66	0.47
Exam of Perioral Areas for Angular Stomatitis/ Cheilosis	Using penlight, inspects around the mouth, corners of the mouth for red cracks/blisters and lips	0.70	−1.83	0.54
Nail Exam for Color, Koilonychia, Beau’s Lines, Splinter Hemorrhage, Clubbing	Inspects nails for nail color, thin, concave, spoon-shaped nails, transverse, deep grooved lines, small splinter like dark hemorrhages, and/or convex clubbed nails with a downward curve	0.92	−0.49	0.54
Self-Introduction	Introduces self and explain the purpose of the exam prior to performing the exam	0.87	0.27	0.11
Verbal Consent	Asks and obtains verbal consent prior to the exam if the patient is alert and oriented	0.87	0.27	0.11
Dry, Brittle Hair/Easily Pluckable Hair	Inspects for dry hair and gently pulls strands of hair to test for brittleness and pluckability	0.83	−1.06	0.53
Patient Dignity	Maintains patient dignity by uncovering patient only as needed, examines all necessary areas when uncovering to minimize exposing patient repeatedly	0.87	0.27	0.11
Eye Conjunctivae for Pale Color	Gently retracts lower eyelids and inspects color using penlight bilaterally	0.86	−0.75	0.53
Skin Exam of Upper & Lower Arm for Follicular Hyperkeratosis, Corkscrew Hair, Lanugo	Inspects skin of upper and lower arm (penlight optional) for papules at tips of hair follicles (rough, goose bump-like appearance), coiled corkscrew or swan-neck shaped hair, or fine, soft hair on arms	0.75	−1.83	0.54
Thenar Eminence	Asks patient to press the pads of thumb and rest of four fingers together, inspects and palpates base of the thumb	0.77	−1.05	0.47
Face & Nasolabial Areas for Flakiness	Inspects nasolabial folds, around nose and corners of the mouth for erythema with scaling	0.75	−1.86	0.54
Oral Ulcer & Lesions	Using penlight inspects for shallow ulcers inside the mouth, base of gums, retracts buccal mucosa to visualize back of the throat, and under the tongue	0.71	−1.60	0.53
Tongue Inspection for Filiform Papillary Atrophy, Magenta/Beefy-Red Tongue, Glossitis	Using penlight and tongue blade, inspects tongue for filiform papillae height, smooth appearance, swelling, or beefy red color	0.59	−2.40	0.52
Gums & Teeth	Using penlight and tongue blade, inspects gums and teeth	0.57	−2.47	0.52
Interosseous Muscles	Asks patient to press the pads of thumb and rest of four fingers together, inspects and palpates muscles near the metacarpal bone	0.72	−1.26	0.44
Patient Interview	Asks patient appropriate questions that help with examination	0.66	−0.47	0.23
Scapular Muscles	Inspects scapula, asks the patient to push against examiner’s hand, and palpates around scapula bilaterally	0.60	−1.72	0.43
Deltoids	Inspects rounded vs squared shoulders, palpates deltoids around shoulders, may ask the patient to raise the arms out to the side to engage the muscle	0.55	−0.72	0.22

* MnSq–Mean Square Residuals, ^ ZSTD–Standardized *z* values.

**Table 3:** Hierarchy of the INSPECT Item Difficulty.

INSPECT Items	Item Performance Indicators	Item Difficulty	Error	INFIT (MnSq) *	ZSTD ^
Handgrip Using Dynamometer When Available (objective measure)	Using standard dynamometer techniques assesses patient’s handgrip strength	4.87	1.71	Maximum	-
Bitot’s Spots	Using penlight, inspects conjunctiva bilaterally for small, foamy, dry, oval, or triangular spots	2.33	0.22	1.03	0.21
Gums & Teeth	Using penlight and tongue blade, inspects gums and teeth	2.06	0.21	0.57	−2.47
Tongue Inspection for Filiform Papillary Atrophy, Magenta/Beefy-Red Tongue, Glossitis	Using penlight and tongue blade, inspects tongue for filiform papillae height, smooth appearance, swelling, or beefy red color	2.02	0.21	0.59	−2.40
Oral Ulcer & Lesions	Using penlight inspects for shallow ulcers inside the mouth, base of gums, retracts buccal mucosa to visualize back of the throat, and under the tongue	1.93	0.21	0.71	−1.60
Eye Conjunctivae for Pale Color	Gently retracts lower eyelids and inspects color using penlight bilaterally	1.84	0.20	0.86	−0.75
Exam of Perioral Areas for Angular Stomatitis/Cheilosis	Using penlight, inspects around the mouth, corners of the mouth for red cracks/blisters and lips	1.77	0.20	0.70	−1.83
Hair Changes	Asks about any recent hair changes, too much hair falling out on the pillow or in the shower?	1.68	0.20	0.96	−0.18
Dry, Brittle Hair/Easily Pluckable Hair	Inspects for dry hair and gently pulls strands of hair to test for brittleness and pluckability	1.60	0.19	0.83	−1.06
Leg Exam for Petechiae and/or Purpura	Inspects for small, pinpoint round skin hemorrhages and/or reddish-purple rash on knees, thighs, or legs	1.53	0.19	1.34	2.04
Nail Exam for Color, Koilonychia, Beau’s Lines, Splinter Hemorrhage, Clubbing	Inspects nails for nail color, thin, concave, spoon-shaped nails, transverse, deep grooved lines, small splinter like dark hemorrhages, and/or convex clubbed nails with a downward curve	1.49	0.19	0.92	−0.49
Skin Exam of Upper & Lower Arm for Follicular Hyperkeratosis, Corkscrew Hair, Lanugo	Inspects skin of upper and lower arm (penlight optional) for papules at tips of hair follicles (rough, goose bump-like appearance), coiled corkscrew or swan-neck shaped hair, or fine, soft hair on arms	1.42	0.18	0.75	−1.83
Face & Nasolabial Areas for Flakiness	Inspects nasolabial folds, around nose and corners of the mouth for erythema with scaling	1.26	0.18	0.75	−1.86
Pitting Edema	Inspects and palpates by pressing down on skin over the pretibial area, ankles, and/or foot for 5 s and releases to assess size and depth of the pit	0.68	0.18	1.25	1.60
Iliac Crest Prominence & Iliac Crest Skinfolds	Inspects tip of the hip bone for prominent iliac crest, pinches skin between the iliac crest and last rib, and rolls to determine the amount of fat to assess fat loss	0.38	0.21	0.94	−0.26
Bilateral Inspection & Palpation	Examines patient bilaterally where applicable	0.35	0.20	0.87	−0.66
Muscles Around Midaxillary Line	Inspects and palpates muscles around the midaxillary line	0.34	0.20	1.29	1.46
Handshake and/or Grip/Squeeze Fingers (subjective measure, not part of Academy/ASPEN diagnostic criteria)	Assesses handgrip strength through handshake and/or asking patient to squeeze examiner’s pointer and middle fingers bilaterally (subjective measure, not part of Academy/ASPEN diagnostic criteria)	0.34	0.22	1.63	2.63
Dentures	Asks if the patient wears dentures and if yes, asks patient if dentures are ill-fitting	−0.03	0.25	1.25	0.96
Thenar Eminence	Asks patient to press the pads of thumb and rest of four fingers together, inspects and palpates base of the thumb	−0.07	0.22	0.77	−1.05
Posterior Trapezius Muscles	Inspects and palpates trapezius on the back bilaterally	−0.15	0.23	0.94	−0.21
Intercostal Muscles	Inspects for concave shape and palpates between ribs for depression	−0.19	0.23	1.0	0.06
Interosseous Muscles	Asks patient to press the pads of thumb and rest of four fingers together, inspects and palpates muscles near the metacarpal bone	−0.25	0.23	0.72	−1.26
Orbital Fat Pads	Inspects eye sockets bilaterally, using pointer and middle fingers, gently palpates around eye sockets	−0.31	0.24	1.52	1.93
Scapular Muscles	Inspects scapula, asks the patient to push against examiner’s hand, and palpates around scapula bilaterally	−0.41	0.26	0.60	−1.72
Pectoralis Major	Asks patient to sit up if able with arms at sides, inspects directly from the front or as feasible, palpates muscles right below clavicles	−0.61	0.28	1.05	0.28
Quadriceps Muscles	With the patient’s leg propped up and slightly bent at the knee, inspects for concave depression, palpates quads bilaterally	−0.77	0.28	1.13	0.51
Gastrocnemius Muscles	With the patient’s leg propped up, gently grasps calf muscle and assesses for bulk or thinning, may ask the patient to flex toes to engage muscle	−0.82	0.29	1.16	0.58
Buccal Fat Pads	Inspects cheeks bilaterally standing directly from the front or as feasible, palpates fat pads under the cheekbone	−0.99	0.32	1.38	1.06
Temporal Muscles	Inspects temporalis muscle directly from the front or as feasible for hollowing/scooping, palpates bilaterally using fingers with patient clenching teeth if able	−1.14	0.34	0.97	0.06
Biceps/Triceps	Asks patient to bend the arm at a 90-degree angle, gently grabs biceps/triceps between thumb and pointer finger and rolls to determine the amount of fat	−1.26	0.36	1.33	0.84
Patient Privacy	Draws curtains, maintains patient’s privacy at all times	−1.55	0.42	1.66	1.25
Patient Interview	Asks patient appropriate questions that help with examination	−1.70	0.47	0.66	−0.47
Deltoids	Inspects rounded vs squared shoulders, palpates deltoids around shoulders, may ask the patient to raise the arm out to the side to engage the muscle	−1.76	0.47	0.55	−0.72
Acromion Process Protrusion	Inspects for prominent protrusion of acromion process	−2.01	0.54	0.75	−0.17
Verbal Consent	Asks and obtains verbal consent prior to the exam if the patient is alert and oriented	−2.98	0.97	0.87	0.27
Self-Introduction	Introduces self and explain the purpose of the exam prior to performing the exam	−2.99	0.97	0.87	0.27
Patient Dignity	Maintains patient dignity by uncovering patient only as needed, examines all necessary areas when uncovering to minimize exposing patient repeatedly	−3.01	0.97	0.87	0.27
Personal Protective Equipment (PPE)	Utilizes appropriate PPE for patients with isolation precautions	−4.01	1.80	Minimum	-
Patient Position	Assures patient comfort, safety and is gentle when moving patient during examination, returns patient’s arms/legs to the original position after completing the exam	−4.15	1.80	Minimum	-
Hand Hygiene	Washes or sanitizes hands prior to patient exam	−4.15	1.80	Minimum	-

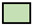
 Micronutrient Items; 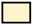 Macronutrient Items; 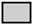 Initial Preparation and Bedside Manner Items; ^ ZSTD—Standardized *z* values, * MnSq—Mean Square Residuals.

**Table 4:** Gaps Between Items.

Items	Gap
Handgrip by Dynamometer and Bitot’s Spot	0.423
Acromion Process and Patient Dignity	0.668
Gums & Teeth and Tongue Exam	1.304
Bitot’s Spot and Gums & Teeth	1.537
Bilateral Palpation and Dentures	3.230
Nasolabial Flakiness and Pitting Edema	3.801
Pitting Edema and Bilateral Palpation	4.366
Patient Dignity and Hand Hygiene	11.833

**Table 5:** INSPECT Person and Item Reliability and Separation Index.

	Reliability Index	Separation Index	Separation Ratio (Ability Levels)
Person	0.86	2.42	3.56
Item	0.96	4.67	-

## Data Availability

The data is not publicly available to protect the confidentiality and privacy of the study participants.
